# Systemic antifungal therapy for proven or suspected invasive candidiasis: the AmarCAND 2 study

**DOI:** 10.1186/s13613-015-0103-7

**Published:** 2016-01-08

**Authors:** Olivier Leroy, Sébastien Bailly, Jean-Pierre Gangneux, Jean-Paul Mira, Patrick Devos, Hervé Dupont, Philippe Montravers, Pierre-François Perrigault, Jean-Michel Constantin, Didier Guillemot, Elie Azoulay, Olivier Lortholary, Caroline Bensoussan, Jean-François Timsit, AmarCAND2 study group

**Affiliations:** Medical ICU, Chatilliez Hospital, Tourcoing, France; U823, Grenoble 1 University, La Tronche, France; Mycology, Rennes University Hospital, Rennes, France; Medical ICU, Cochin University Hospital, Paris, France; Bio Statistics Unit, Lille University Hospital, Lille, France; Surgical ICU, Amiens University Hospital, Amiens, France; Anesthesiology and Critical Care Medicine, Bichat-Claude Bernard University Hospital, Paris, France; Medical-surgical ICU, Montpellier University Hospital, Montpellier, France; Perioperative Medicine Department, Clermont-Ferrand University Hospital, Clermont-Ferrand, France; Unité de Pharmaco-épidémiologie et Maladies Infectieuses, Institut Pasteur, Paris, France; Medical ICU, Saint-Louis University Hospital, Paris, France; Necker Pasteur Center for Infectious Diseases, Necker Enfants-Malades Hospital, Paris, France; Medical Affairs, MSD France, Courbevoie, France; Medical ICU, Bichat-Claude Bernard University Hospital, Paris, France

**Keywords:** *Candida*, Intensive care, Cohort study, Candidiasis, Candidaemia, Critically ill

## Abstract

**Background:**

In the context of recent guidelines on invasive candidiasis (IC), how French intensive care units (ICUs) are managing IC?

**Methods:**

This is a prospective observational multicenter cohort study. During 1 year (2012–2013), 87 French ICUs enrolled consecutive patients with suspected or proven IC (SIC or PIC) and receiving systemic antifungal therapy (SAT). Data were collected up to 28 days after inclusion.

**Results:**

We studied 835 patients, 291 with PIC and 544 with SIC. At SAT initiation, patients with SIC were significantly more severe (SAPS II 50.1 ± 18.7 vs. 46.2 ± 18.0). Severe sepsis or septic shock prompted to initiate empiric SAT in 70 % of SIC. Within 4 days in median, the initial SAT was modified in 49 % of patients with PIC vs. 33 % patients with SIC. Modifications were most often motivated by mycological results, and de-escalation was the most frequent change. Regarding compliance to IC management guidelines, echinocandin was used for 182 (62.5 %) patients with PIC, and 287 (52.7 %) of those with SIC; central venous catheter was removed in 87 (54.3 %) of patients with candidaemia, and 43 of the remaining patients received echinocandin; and de-escalation was undertaken after 5 days of SAT in 142 patients, after 10 days in 13 patients. As 20.6 % of SIC were secondarily documented, 403/835 (48 %) patients had finally a proven IC. *Candida albicans* was the main pathogen (65.3 %), then *Candida glabrata* (15.9 %). The 28-day mortality rates were 40.0 % in candidaemia, 25.4 % in cIAI, and 26.7 % in deep-seated candidiasis. In the overall population of patients with proven IC, four independent prognostic factors were identified: immunosuppression (Odds Ratio (OR) = 1.977: 1.03–3.794 95 % confidence interval (CI), p = 0.04), age (OR = 1.035; 1.017–1.053 95 % CI; *p* < 0.001), SAPS >46 on ICU admission (OR = 2.894; 1.81–4.626 95 % CI; *p* < 0.001), and surgery just before or during ICU stay (OR = 0.473; 0.29–0.77 95 % CI; *p* < 0.001).

**Conclusion:**

When SAT is initiated in French ICUs, the IC is ultimately proven for 48 % of patients. Empiric SAT is initiated in severely ill ICU patients. The initial SAT is often adapted, with de-escalation to fluconazole when possible. Mortality rate remains high.

**Electronic supplementary material:**

The online version of this article (doi:10.1186/s13613-015-0103-7) contains supplementary material, which is available to authorized users.

## Background

The management of critically ill patients in intensive care units (ICUs) has improved during the last decades yielding increased survival of patients with complex medical and surgical issues. Such patients are at high risk of invasive infections, including invasive candidiasis (IC) [1, 2]. IC can involve any organ; bloodstream infections, complicated intra-abdominal infections (cIAI), and deep-seated infection are the most frequently diagnosed [3, 4]. In the EUROBACT study, fungal bloodstream infections represented 7.8 % of monomicrobial infections [5]. The mortality rate of IC remains high. In the EPIC II study, mortality rate of candidaemia was 42.6 % [2].

Diagnosing IC remains difficult, and requires often some days [3, 6, 7]; in addition, blood cultures have insufficient diagnostic accuracy [4, 8, 9]. Of note, while some studies showed a delayed administration of systemic antifungal therapy (SAT) was associated with increased mortality [10–12], others did not found any relationship between prognosis and the timing of initial therapy [13, 14]. Therefore, SAT is often initiated empirically, despite the lack of consensus on decision-making criteria [3, 15]. Some scores based on risk factors-based predictions have been set to help clinicians [16–18]; however, their positive predictive value remains insufficient.

As per IDSA and ESCMID guidelines, an appropriate management of IC includes the administration of the appropriate SAT [3, 4]. For suspected invasive candidiasis (SIC) as for proven candidaemia, IDSA recommends to use fluconazole or an echinocandin, preferably echinocandin for more severe patients, whereas ESCMID guidelines do not modulate their recommendations according to patient severity.

This prospective study intended to describe the management of IC in French ICUs, including the epidemiology of the *Candida* species involved and the outcome of the patients.

## Methods

### Study design and objectives

AmarCAND2 is a multicenter, prospective, observational study, involving French ICUs having managed at least one IC within the past year. Most centers were involved in the previous AmarCAND study [19].

The study primarily aimed to describe the prescribing practices for SAT in ICUs. SAT could be targeted for patient with microbiologically proven invasive candidiasis (PIC), or empirically instituted for SIC. The secondary objectives included the analysis of the *Candida* species involved in the IC, and of the patient outcome according to the final IC diagnosis, and finally of prognostic factors associated with mortality of the overall population of patients with a definite diagnosis of IC. In addition, the level of compliance of the participating ICUs with the ESCMID guidelines (at draft stage when the study was conducted) and with the IDSA guidelines was analyzed. With respect to IDSA guidelines, the use of echinocandin for severely ill patients, an appropriate source control (e.g., central venous catheter (CVC) removal in case of candidaemia), and de-escalation for fluconazole-susceptible strains when the patient is stabilized were evaluated. Regarding ESCMID guidelines, the compliance with the following recommendations was evaluated: a preferred use of echinocandin, the removal of any CVC in case of candidaemia (if not possible, echinocandin or liposomal amphotericin B use is recommended), and de-escalation to be considered after 10 days of treatment for candidaemia.

The study was designed and overseen by an independent Scientific Committee, and coordinated by a clinical research organization. Investigators enrolled patients according to the study protocol and managed them according to their own clinical judgment. The Ethics Committee of the French Intensive Care Society and the French National Committee for Data Protection and Freedom of Information approved the study. According to French regulations, such an observational study does not require patients to sign an informed consent.

### Patients

Investigators enrolled adult patients requiring SAT for PIC or SIC, during their ICU stay (≥48 h post-admission), except patients with recent (<15 days) solid organ transplant, or neutropenia (absolute neutrophil count <1 × 10^12^ cells/L), or those receiving SAT prophylactically or for a mold infection.

### Clinical and mycological data collection and definition

Data were collected using an electronic case report form. At the initiation of SAT, the patient was enrolled, and data were collected (Additional file 1: Clinical data collection).

At the time of SAT initiation, patients were divided into two groups. Patients for whom the presence of a positive microbiologic specimen prompted initiation of SAT were included in the PIC group. Patients for whom only a suspicion of IC led to initiate SAT were included in the SIC group. Nevertheless, in this later group, patients may ultimately have a documented IC if appropriate samples drawn before SAT initiation yielded *Candida* spp.

Documented IC was defined as candidaemia, cIAI, or deep-seated candidiasis. Candidaemia was defined by the presence of ≥1 positive blood culture for *Candida* spp. A positive microscopic examination or culture for *Candida* spp. from a peritoneal sample drawn during surgery or percutaneously, excluding drain samples, defined cIAI. Finally, deep-seated candidiasis was defined by a positive specimen from a deep organ or fluid usually sterile (except urine and respiratory secretions).

### Mycological tests

The mycology results provided by the laboratory of each hospital enabled patients to be classified as candidaemia, cIAI, deep-seated infection, or not proven infection. Isolates were classified as susceptible, susceptible-dose dependent, or resistant to antifungals, using various methods and according to the manufacturers’ recommendations. In France, the main marketed methods used in routine laboratories refer to CLSI interpretive categories (http://www.clsi.org). E-test method (BioMérieux, France) was used in 75 % of the centers.

### Modification of initial antifungal treatment

In case of initial SAT modification, the investigator was asked to record the date and reason(s) for the modification. If a new SAT was started, the antifungal agent was recorded.

### Patient outcome

At the end of the SAT, the *Candida* infection outcome, the date of ICU discharge, and the vital status of all patients at that date and at day 28 were collected.

### Statistical analyses

Statistical analyses were performed using SAS (version 9.3). Variables were expressed as mean with standard deviation for numerical variables and as frequencies and percentages for categorical variables. Groups were compared using Wilcoxon, Chi-square, or Fisher’s exact tests, as appropriate, with a statistical significance threshold of 0.05.

A stepwise logistic regression analysis was performed to identify risk factors associated with mortality on day-28, regardless of its cause. In order to identify independent risk factors for mortality, variables were included in the multivariate model if the *P* value was ≤0.20 in bivariate analysis. Adjusted Odds ratios (AOR) were computed using logistic regression analysis including the independent predictors of mortality.

All statistical analyses were performed using SAS software, V9.1.

## Results

**Fig. 1:**
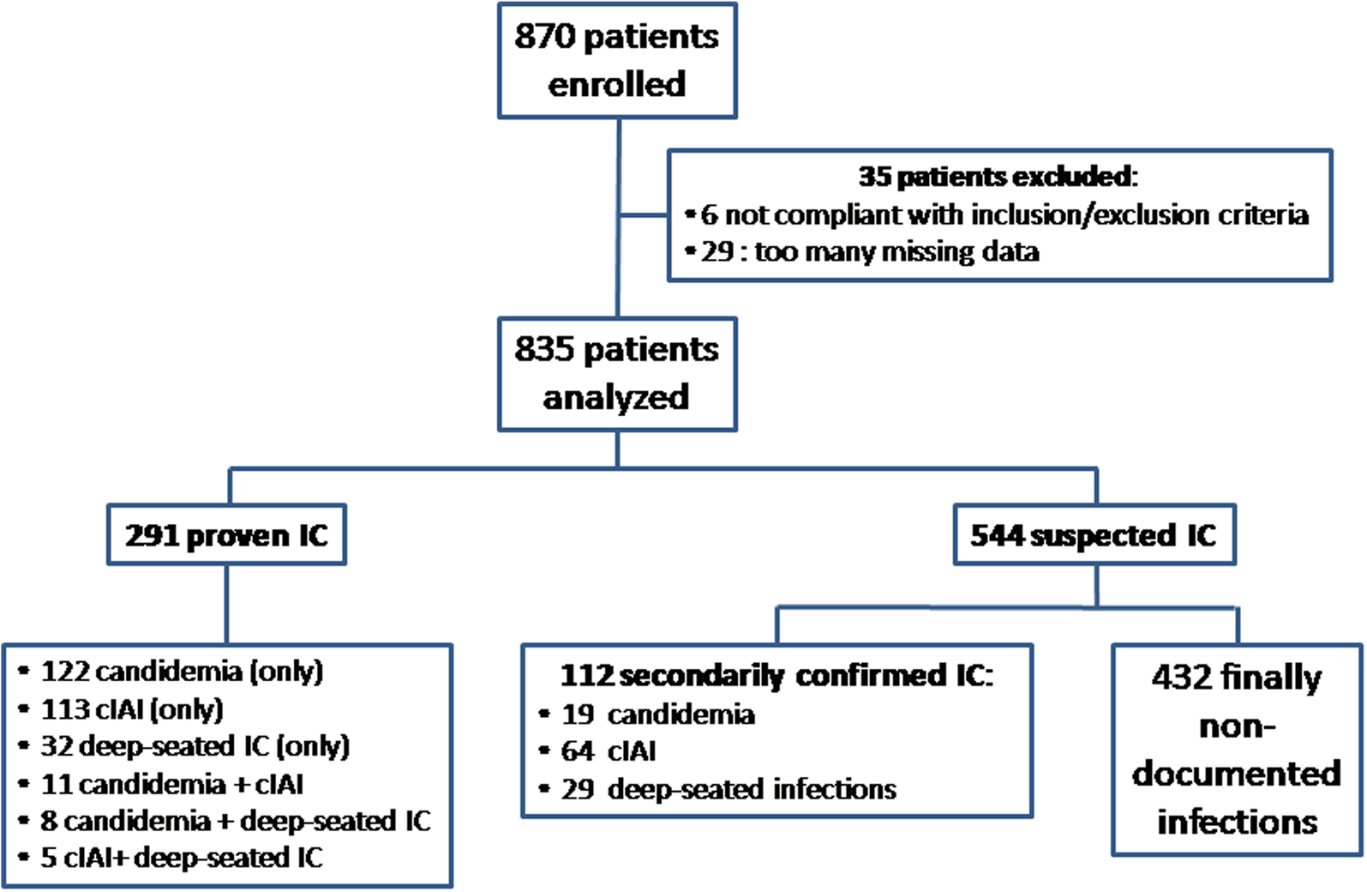
Disposition of patients enrolled and analyzed. *IC* invasive candidiasis, *cIAI* complicated intra-abdominal infections

From Oct. 2012 to Oct. 2013, 87 ICUs enrolled 870 patients (Fig. 1), of whom 35 were excluded for non-respect of inclusion criteria (*n* = 6) and for missing data (*n* = 29). So, we studied 835 patients, 291 with PIC when SAT was started and 544 for whom SAT was started for SIC. Among the 291 patients with PIC at SAT initiation, candidaemia, cIAI, and deep-seated infections were diagnosed in 141, 129, and 45 patients, respectively (24 had infections at multiple sites).

Characteristics of patients at the ICU admission are listed in the Additional file 1: Table E1. Characteristics at SAT initiation are described in Table 1. Centers which enrolled more than 22 patients enrolled more SIC compared to PIC; otherwise centers did not differ significantly for the rate of PIC and SIC enrolled (Additional file 1: Table E2).

**Table 1:** Characteristics of patients with initially proven and suspected invasive candidiasis

Patients’ characteristics	SAT for initially proven invasive candidiasis *N* = 291	SAT for suspected invasive candidiasis *N* = 544	*P* value
At ICU admission			
Age (years)	62.2 ± 15.4	61.9 ± 14.5	0.93
Male gender	185 (63.6)	337 (62.0)	0.64
Reason for admission to ICU			
Medical	119 (40.9)	268 (49.3)	0.02
Surgical	161 (55.3)	260 (47.8)	0.04
Trauma	16 (5.5)	16 (2.9)	0.08
C-reactive protein (mg/L)	165.9 ± 109.4	173.1 ± 122.6	0.67
Creatinine (µmol/L)	145.8 ± 109.6	132.1 ± 96.9	0.13
Red blood cell transfusion in ICU	163 (56.0)	289 (53.1)	0.42
Platelet transfusion in ICU	60 (20.6)	119 (21.9)	0.67
At SAT initiation			
Length of hospital stay before SAT (days)	13 [6; 23]	11 [4; 20]	<0.01
Length of ICU stay before SAT (days)	5 [2; 14]	4 [0; 11]	<0.01
Body temperature (°C)	37.6 ± 1.3	37.7 ± 1.3	0.34
SAPS II	46.2 ± 18.0	50.1 ± 18.7	0.006
SOFA score	7.3 ± 4.8	8.3 ± 4.3	0.0007
Septic shock	120 (41.2)	330 (66.7)	<0.0001
Severe sepsis	118 (40.6)	203 (37.3)	0.36
Risk factors for invasive candidiasis^a^			
Invasive mechanical ventilation	196 (67.4)	458 (84.2)	<0.0001
Central venous catheter	278 (95.5)	526 (96.7)	0.44
Urinary catheterization	280 (96.2)	516 (94.9)	0.50
Antibiotic treatment	268 (92.1)	481 (88.4)	0.12
Total parenteral nutrition	149 (51.2)	235 (43.2)	0.03
Hemodialysis or hemodiafiltration	92 (31.6)	160 (29.4)	0.51
Corticosteroid treatment	55 (18.9)	151 (27.8)	0.005
Surgery just before or during ICU stay	197 (67.7)	341 (62.7)	0.15
Abdominal surgery	156 (78.2)	263 (79.2)	0.80
Scheduled abdominal surgery	16 (10.3)	48 (18.3)	0.03
Emergency abdominal surgery	140 (89.7)	215 (81.7)	0.03
Mortality on D28	96 (34.4 %)	163 (31.6 %)	0.42

The results are given as n (%) or mean ± SD, when indicated

*SAT* systemic antifungal therapy, *ICU* intensive care unit, *SOFA* sequential organ failure assessment; SAPS II: simplified acute physiology score

^a^Risk factors as per IDSA and ESCMID guidelines (3, 4)

In the SIC group, the clinical and microbiological features taken into account for initiating empiric SAT are reported in Additional file 1: Table E3. Of note, investigators reported that 70.4 % patients had a septic shock, 50.9 % a recent surgery, and 36.0 % a persistent fever. For 54.6 % of patients with empiric SAT, 4 to 8 features were reported (and from 2 to 10 features for 75 % of patients); up to 14 features were reported.

The initial SAT was based on a single agent for most patients (Table 2; Additional file 1: Doses of antifungal agents). Median doses of antifungal agents are reported in additionnal file 1. The text in additional file was as follows: At SAT initiation, the median doses of fluconazole, voriconazole, caspofungin and micafungin were 800 mg, 800 mg, 70 mg and 100 mg, respectively. On days 2 to 4, doses were 400 mg, 400 mg, 50 mg and 100 mg, respectively. The comparison of patients according to the SAT type, echinocandins vs. azoles, showed that patients receiving an echinocandin had higher severity scores both on ICU admission and at the time of SAT initiation, and a higher rate of septic shock at SAT initiation (Additional file 1: Table E4, Factors associated with echinocandin prescription).Addtional file 1. The following text could be related to this insert: A multivariate analysis was conducted to analyze the factors associated with echinocandin prescription. Variables with a p-value of 0.2 in an univariate analysis were introduced in a multivariate logistic regression. The selected variables were the followings: (1) at ICU admission: SAPSII, gender, BMI, presence of comorbidities, type of admission (medical reasons vs. other), surgery before ICU admission, interval from hospitalization; (2) at SAT initiation: parenteral nutrition, presence of bacteria, septic shock, antibacterial therapy, red blood cell transfusion, creatinine, central venous catheter, proven IC (vs. suspected). The final model showed that a proven invasive candidiasis, septic shock, SAPSII score greater than 46, ICU admission for medical reasons, a creatinine greater than 103 µmol/L, and the presence of a central venous catheter increased significantly the probability of echinocandin prescription (see Table E4).

**Table 2:** Summary of initial antifungal treatment in patients with proven and suspected invasive candidiasis

Antifungal treatment	SAT for proven invasive candidiasis *N* = 291	SAT for suspected invasive candidiasis *N* = 544
Monotherapy	287 (98.6 %)	541 (99.5 %)
Polyenes	6 (2.1 %)	6 (1.1 %)
Amphotericin B deoxycholate	0	3
Liposomal amphotericin B	4	3
Amphotericin B lipid complex	2	0
Azoles	99 (34.5 %)	248 (45.8 %)
Fluconazole	99	244
Voriconazole	0	4
Echinocandin	182 (63.4 %)	287 (53.0 %)
Caspofungin	160	252
Micafungin	20	34
Anidulafungin	0	1
Flucytosine	0	0
Combination therapy	4	3
Liposomal amphotericin B and flucytosine	0	2
Caspofungin and fluconazole	2	0
Caspofungin and voriconazole	1	0
Caspofungin and flucytosine	1	0
Amphotericin B deoxycholate and micafungin	0	1

The results are given as *n* (%)

*SAT* systemic antifungal therapy

Overall, 325 isolates were identified among the 291 patients with PIC (Table 3). *Candida albicans* was the most common pathogen. *Candida* spp. were associated with bacterial pathogens in 19/141 patients with candidaemia and 76/129 patients with cIAI. Among the 544 patients with SIC, IC was ultimately proven for 112 for whom 128 isolates were identified (Fig. 1). Again, *C. albicans* was the most common pathogen (Table 3). The comparison of patients with initially proven IC (*n* = 291) vs. patients with ultimately proven IC (*n* = 112) shows the following significant differences (Additional file 1: Table E5): on ICU admission, patients with initially proven IC had a higher SOFA score; at SAT initiation, patients with ultimately proven IC were more often mechanically ventilated, had undergone more often surgery before or during ICU stay, and exhibited more often septic shock.

**Table 3:** Results of mycological cultures of samples from the AmarCAND2 patients, according to their status at SAT initiation: invasive candidiasis either proven, or suspected

	SAT for proven invasive candidiasis *N* = 291	SAT for suspected invasive candidiasis *N* = 544	Overall population *N* = 835
Patients with at least an isolate identified, i.e., proven IC	291 (100)	112 (20.6)	403 (48.3 %)
Number of identified isolates	325	128	453
*C. albicans*	206 (63.4)	90 (70.3)	296 (65.3)
*C. glabrata*	54 (16.6)	18 (14.0)	72 (15.9)
*C. parapsilosis*	19 (5.8)	2 (1.6)	21 (4.6)
*C. tropicalis*	14 (4.3)	6 (4.7)	20 (4.4)
*C. krusei*	10	3	13
*C. lusitaniae*	9	1	10
*C. kefyr*	6	2	8
*C. guilliermondii*	3	1	4
*C. dubliniensis*	2	0	2
*C. norvengiensis*	1	1	2
*C. pulcherina*	1	0	1
Other	0	4	4

The results are given as *n* (%)

*SAT* systemic antifungal therapy, *IC* invasive candidiasis

In the overall population, IC was thus finally proven for 403/835 patients (Fig. 1). Among patients with deep-seated IC (*n* = 74), infections mainly involved pleura (*n* = 22), hepato-biliary-pancreatic tract (*n* = 14), bone or joints (*n* = 4), skin and soft tissues (*n* = 4), mediastinal space (*n* = 4), and heart valve or vascular prosthesis (*n* = 3). The comparison of patients with proven IC (*n* = 403) with patients without IC (*n* = 432) is reported in Additional file 1: Table E6. At ICU admission, patients with IC exhibited a significant lower SAPS II. At SAT initiation, patients with IC exhibited a lower SOFA score, and had less often septic shock, invasive mechanical ventilation, and previous corticosteroid treatment, whereas surgery just before or during ICU stay was more often present.

The median time elapsed between sample draw and mycological results was 1 day for the positivity to yeasts, 3–4 days for the species identification, and 5–6 days for the antifungal susceptibility. Local laboratory MIC50 (number of isolates; IQR) for the main antifungals tested was as follows: fluconazole, 0.25 mg/L (*n* = 283; 0.125–1.50), voriconazole, 0.02 mg/L (*n* = 228; 0.01–0.06), caspofungin, 0.09 mg/L (*n* = 253; 0.05–0.125), and micafungin, 0.02 mg/L (*n* = 74; 0.01–0.03). The overall rate resistance among all isolates was 13 % for fluconazole (including 20.3 % among *C.* *glabrata*), 2 % for voriconazole, 1.25 % for micafungin, and 0.7 % for caspofungin.

The initial SAT was modified for 142 (48.8 %) patients with PIC, 4.77 ± 4.01 days after initiation. The changes were motivated by mycological documentation in 118 patients. They were largely for selecting another antifungal drug (139/142). Main modifications were de-escalation from an echinocandin to fluconazole for 93 patients, and escalation from fluconazole to an echinocandin for 20 patients. The initial SAT was terminated or modified for 180 (33.1 %) patients with SIC, 5.37 ± 5.25 days after initiation. SAT was terminated in 76 patients (confirmation of the bacterial origin of the infection) and was modified in 104, mainly according to mycological documentation. De-escalation from an echinocandin to fluconazole was performed in 60 patients, and escalation from fluconazole to an echinocandin occurred in 30.

With regard to source control, a surgical procedure (*n* = 191) or a percutaneous drainage of intra-abdominal abscess (*n* = 2) was performed in all patients with cIAI. CVC was removed in 87 (54.3 %) patients with candidaemia. This removal occurred 2 days or later after blood sampling identifying candidaemia in 34.4 % of cases. Among the 73 patients without CVC removal, 6 died before the result of blood sampling identifying *Candida* spp. was known, and 5 had no CVC. Among the 62 remaining patients, 43 patients received an echinocandin.

Compliance to three main recommendations of IDSA guidelines was analyzed; (1) echinocandin was used for 182 (62.5 %) of patients with PIC, and 287 (52.7 %) of patients with SIC; (2) CVC was removed in 87 (54.3 %) patients with candidaemia; (3) de-escalation was undertaken 5 days after SAT initiation for 142 (17 %) patients. At least one recommendation was applied for 506 patients. With regard to the compliance to the similar recommendations of ESCMID guidelines, (1) echinocandin was used for 182 (62.5 %) of patients with PIC, and 287 (52.7 %) of patients with SIC; (2) CVC was removed in 87 (54.3 %) patients with candidaemia. Among the 62 patients with CVC and mycological results known while still alive, 43 (69.3 %) were treated with echinocandin; (3) 10 days after SAT initiation, among the 484 patients still alive in ICU, de-escalation was undertaken for 13 (2.7 %) patients. At least one recommendation was applied for 505 (60.5 %) patients.

The clinical outcome of the patients according to their final diagnosis and SAT duration is depicted in Table 4. Cure rates ranged from 55 to 70 %. The mortality rates were 40.0 % in candidaemia, 25.4 % in cIAI, and 26.7 % in deep-seated candidiasis. In patients with vs. without immunodeficiency, mortality rates were 58.3 vs. 37.3 % (*p* = 0.11), 40 vs. 23.65 % (*p* = 0.11), and 30 vs. 25 % (*p* = 0.71), respectively. Among patients with candidaemia, the mortality rate was 38 % in patients with isolated candidaemia (*n* = 54/141), 45 % in patients with candidaemia associated with cIAI (*n* = 5/11), and 62.5 % in patients with candidaemia associated with deep-seated candidiasis (*n* = 5/8) (*p* = 0.4). Among patients with candidaemia and non-immunosuppressed (*n* = 134), the mortality rate was 37.3 % (50/134). It was not significantly different according to the removal of the CVC (35 vs. 40 %; *p* = 0.59), to the early SAT initiation, i.e., before the knowledge of positive blood culture (31 vs. 38 %; *p* = 0.61), or to the use of echinocandin as SAT (39 vs. 31 %; *p* = 0.42).

**Table 4:** Clinical evolution as a function of the final diagnosis of invasive candidiasis

	Final diagnosis			
	Candidaemia *n* = 160	CIAI *n* = 193	Deep-seated candidiasis *n* = 74	Non-confirmed IC *n* = 432
SOFA score at D0				
Mean ± SD	7.7 ± 4.8	7.2 ± 4.5	7.7 ± 4.5	8.4 ± 4.3
Median (min; max)	7 (0; 23)	7 (0; 19)	7.5 (0; 17)	8 (0; 24)
SOFA score at D7				
Mean ± SD	6.9 ± 6.0	4.9 ± 5.1	6.5 ± 5.8	6.8 ± 5.3
Median (min; max)	5 (0; 24)	3 (0; 24)	5 (0; 21)	6 (0; 24)
Clinical outcome *n* (%)				
Cured	89 (55.6)	135 (70.0)	48 (64.9)	261 (60.4)
Failure	27 (16.85)	20 (10.35)	12 (16.2)	42 (9.7)
Not determined	33 (20.6)	27 (14.0)	13 (17.6)	89 (20.6)
Information not available	11 (6.9)	11 (5.7)	1 (1.3)	40 (9.3)
Status on D28, *n* (%)				
Alive	90 (56.3)	135 (70.0)	53 (71.6)	271 (62.7)
Dead	64 (40.0)	49 (25.4)	19 (26.7)	138 (32.0)
Lost to follow-up after ICU discharge	6 (3.7)	9 (4.6)	2 (2.7)	23 (5.3)
Length of SAT among those alive (days)				
Mean ± SD	24 ± 16	20 ± 14	28 ± 25	12 ± 10
Median	20	17	21	10

*cIAI* complicated intra-abdominal infections, *IC* invasive candidiasis, *SAT* systemic antifungal therapy, *SD* standard deviation

In the overall population of patients with proven IC (*n* = 403), 121 patients died. Factors associated with 28-day mortality are reported in Table 5. In multivariate analysis, four independent prognostic factors were identified: immunosuppression (Odds Ratio (OR) = 1.977: 1.03–3.794 95 % confidence interval (CI), *p* = 0.04), age (OR = 1.035; 1.017–1.053 95 % CI; *p* < 0.001), SAPS >46 on ICU admission (OR = 2.894; 1.81–4.626 95 % CI; *p* < 0.001), and surgery just before or during ICU stay (OR = 0.473; 0.29–0.77 95 % CI; *p* < 0.001). Of note, early SAT instituted before the knowledge of positive sample did not influence outcome (OR = 1.507: 0.891–2.548 95 % CI; *p* = 0.13).

**Table 5:** Factors associated with 28-day mortality in patients with proven IC

Patients’ characteristics	Survivors (*N* = 273)	Non survivors (*N* = 130)	*P* value
At ICU admission			
Age	61.5 [51; 70.1]	66.5 [57.9; 78.8]	<.01
Male gender	171 (62.6)	83 (63.8)	0.81
SAPSII	42 [30; 52]	56 [45; 67]	<0.01
SOFA score	8 [5; 10]	9.5 [7; 13]	<0.01
Immunosuppression	29 (10.6)	31 (23.8)	<0.01
Reason for admission to ICU			<0.01
Medical	88 (32.2)	66 (50.8)	
Elective surgical	24 (8.8)	14 (10.8)	
Emergency surgical	144 (52.7)	48 (36.9)	
Trauma	17 (6.2)	2 (1.5)	
Red blood cell transfusion in ICU	141 (51.6)	79 (60.8)	0.09
Platelet transfusion in ICU	49 (17.9)	36 (27.7)	0.03
At SAT initiation			
Body temperature (°C)	38 [37; 38.5]	37.6 [36.9; 38.2]	<0.01
SOFA score	6 [3; 9]	9 [6; 13]	<0.01
Septic shock	110 (40.3)	72 (55.4)	<0.01
Severe sepsis	117 (42.9)	48 (36.9)	0.26
Invasive mechanical ventilation	191 (70)	97 (74.6)	0.33
Central venous catheter	261 (95.6)	126 (96.9)	0.53
Urinary catheterization	259 (94.9)	129 (99.2)	0.03
Hemodialysis or hemodiafiltration	68 (24.9)	53 (40.8)	<0.01
Total parenteral nutrition	135 (49.5)	64 (49.2)	0.97
Corticosteroid treatment	44 (16.1)	38 (29.2)	<0.01
Surgery just before or during ICU stay	209 (76.6)	75 (57.7)	<0.01
Initial SAT			0.26
Amphotericin B	4 (1.5)	2 (1.5)	
Fluconazole	112 (41)	40 (30.8)	
Voriconazole	2 (0.7)	1 (0.8)	
Echinocandins	155 (56.8)	87 (66.9)	
SAT instituted before the knowledge of positive sample			0.14
No	191 (70)	100 (76.9)	
Yes	82 (30)	30 (23.1)	

The results are given as *n*(%) or median (min; max), when indicated

*SAT* systemic antifungal therapy, *ICU* intensive care unit, *SOFA* sequential organ failure assessment, *SAPS II* simplified acute physiology score

## Discussion

This study on SAT in French ICUs enrolled 291 patients with PIC and 544 with SIC. Patients receiving SAT for SIC were more severely ill than those having SAT for PIC: they had higher severity scores and more often septic shock. They had more often known IC risk factors. Initial SAT was mainly based on echinocandins. However, in case of adaptation, fluconazole was mainly chosen. Mortality remains high, particularly for candidaemia (40.0 %).

The appropriate IC management includes the administration of the right antifungal agent [3, 4]. Regarding comparable population set within the previous AmarCAND study, caspofungin was administered to 18.1 % of patients, while 72.7 % received fluconazole [19]. In contrast, at SAT initiation for AmarCAND2, caspofungin was the most frequent agent administered. It was significantly preferred for more severe patients, for targeted as for empiric SAT. ESCMID guidelines recommend treating patients with suspected IC with echinocandin [3], similarly to IDSA guidelines [4], although the latter recommends using echinocandin for severely ill patients. Our study patients were admitted in ICU, thus severely ill, which is consistent with the rather high rate of patients treated with echinocandin (63.4 % of PIC patients, 53.0 % of SIC patients). Indeed, echinocandin were significantly more often administered in case of illness severity such as septic shock or high severity scores. Another recommendation of both guidelines which was evaluated relates to a careful control of source. Most, but not all, patients with candidaemia had CVC, which was removed in one patient out of two. This rate is not very high; however, IDSA and ESCMID recommendations differ slightly. IDSA guidelines recommend CVC removal, whereas ESCMID guidelines add that patients whom CVC cannot be removed should be treated with echinocandin or liposomal amphotericin B. Indeed, among patients with candidaemia and CVC not removed, most patients (69.3 %) received echinocandin. For patients with cIAI, the source control is abdominal surgery or at least peritoneal drainage, which was undertaken for all, as it was a study inclusion criterion. The last recommendation of both guidelines which was evaluated relates to the de-escalation of SAT. According to IDSA guidelines, de-escalation should be performed in case of IC due to fluconazole-susceptible strains when the patient becomes stable, whereas ESCMID guidelines recommend de-escalation after ten days of intravenous SAT for patients with candidaemia. Overall, regarding de-escalation, IDSA recommendation was applied for 17 % of all patients. The compliance rate with IDSA or ESCMID guidelines is rather poor. However, although ESCMID guidelines were circulated through 2011 ECCMID workshop slides, they were officially issued after the study start. Regarding the duration of SAT, it was shorter among patients whose IC was eventually not documented than among patients with proven IC, and similar to that observed in the FongiDay study: 11.7 days on average and no longer than 16 days [20].

In our study, *C. albicans* remained the leading *Candida* species, followed by *C.* *glabrata,* which is the leading non-*albicans* pathogen in France [19, 21], in Northern European countries [22–24], and in the US [11, 25]. This rather low rate of *C. glabrata,* close to 16 % in our study, explains why fluconazole is chosen in half of patients. This rate is comparable to that observed during the previous AmarCAND study [19]. It remained stable despite the use of echinocandins, in contrast with the results of a recent French study showing a higher rate of *C. glabrata* (20.0 %), increasing after previous exposure to caspofungin [26]. Regarding antifungal susceptibility, antifungal resistance was uncommon except for 13 % of isolates classified as fluconazole-resistant.

Mycological cultures require some days for the full process from the sampling to the susceptibility results. Mycological results were the main drivers for treatment modifications, which occurred in almost half of the targeted SATs. Empiric SAT was modified in one-third of patient, more often based on mycological results than on clinical changes. In 42 % patients whom treatment was modified, the SAT was terminated based on positive bacteriological results combined with negative mycological tests results. For empiric as for targeted SAT, the most frequent initial drug was caspofungin, and the most frequent agent modification was de-escalation to fluconazole. The delayed availability of mycological results likely explains the high rate of patients with empiric SAT [27]. Other laboratory tests, not based on cultures, have been developed, such as *Candida*-specific polymerase-chain-reaction, or tests based on the detection of yeasts antigens, or of antibodies directed against these antigens. In our study, the use of non-culture-based diagnostic methods to suspect IC and so to begin SAT was still anecdotal (less than 4 %). These tests are now recommended by the ESCMID guidelines for the IC diagnosis [8], and will probably be increasingly implemented as far as robust and standardized methods will be available.

The identification of high-risk patients who may benefit from empiric SAT is still challenging. Major risk factors for IC are well known [3, 4, 7, 28]; however, they are so common in ICU patients than other tools are needed. The *Candida* colonization index and the *Candida* score are useful for their good negative predictive value enabling to identify patients at low risk of IC [16, 18]. Whether the study investigators used these scores was not captured. Nevertheless, it could be suggested that, in this study, the overall conditions leading investigators to initiate empiric SAT had both low specificity since a minority of patients with empiric SAT had a secondarily proven IC (112/544) and low sensitivity since only a minority of patients with proven IC benefited from an early SAT (19/141 candidaemia, 64/129 cIAI, and 29/45 deep-seated infections). These rates are disappointing compared to results showed by Bruyere et al. in a study on ICU patients with suspected IC based on uncontrolled sepsis despite at least one 2-day course of antibacterial agents: the use of the *Candida* score with a cut-off point at 3 or more triggering SAT prescription for these patients was associated with sensitivity, specificity, positive and negative value of 69.2, 82.1, 69.2, and 82.1 %, respectively [29]. However, the rate of secondarily proven IC among those empirically treated (20.4 %) likely reflects the specific interest of participating ICUs regarding invasive candidiasis risk assessment.

IC remains associated with a high mortality rate. In our study, we observed a day-28 mortality rate set at 40, 25.7, and 25.4 % for candidaemia, deep-seated IC, and cIAI, respectively. Such a result is in accordance with literature data. In a nationwide US study collecting data on bloodstream infections from 1995 to 2002, the crude mortality rate of candidaemia among ICU patients reached 47 % [30]. In AmarCAND1 study, the 30-day mortality was 45.9 % in patients with PIC [19]. Finally, in a study conducted from 2002 to 2010 among ICU patients with candidaemia, the day-28 mortality rate increased from 41.5 to 56.9 % [26]. Among risk factors of poor prognosis identified in this study, a greater age, immunodeficiency, and SAPSII >46 are well-known risk factors of poor prognosis in ICU patients overall. A recent surgery was not previously identified as a protective factor for IC prognosis. However, the surgical origin of ICU patients was already identified as a factor of good prognosis for candidaemia [31].

This study had some limitations. First, it was conducted in one country and results could not fully be applied to other countries. However, it involved a large number of ICUs with a broad case-mix range of medical and surgical patients, and provides an accurate picture of the SAT practices. Second, it was an observational study, without any study-specific intervention; however, it is the first study comparing the management of PIC and SIC within the same establishments. Third, microbiological methods used to determine MIC were different in the participant hospitals, leading to interpret these results with caution. Finally, fungal infection management was likely a field of interest for most investigators; therefore, physicians were more likely to know and follow the guidelines and to administrate properly the SAT. In addition, we cannot ensure that absolutely all consecutive patients receiving SAT were included in the study. Therefore, it might hamper the extrapolation of these study results to other ICUs.

## Conclusion

This large prospective observational study conducted showed the management of IC and the strategy of SAT use in French ICUs. Almost half of patients had an ultimately proven IC, of whom only 27 % received early SAT before mycological documentation. This later point could explain why the mortality associated to IC remained high, particularly in patients with candidaemia. ESCMID guidelines were not officially issued at the time our study was conducted, and there are some discrepancies between the IDSA and the ESCMID recommendations we evaluated, which altogether might have contributed to the rather low rate of compliance observed in this study. Comparatively to AmarCAND1, the diversity of the *Candida* species was maintained, the rate of IC due to *C. albicans* remained high, and the rate of *C. glabrata* remained stable. Further analyses will be conducted to further describe epidemiological and mycological study results.

## Additional file

10.1186/s13613-015-0103-7 **Table E1.** Characteristics of patients with proven and suspected invasive candidiasis at ICU admission. **Table E2.** Rate of proven versus suspected invasive candidiasis enrolled by center according to center characteristics. **Table E3.** Among the 544 ICU patients with SAT for suspicion of invasive candidiasis, characteristics of patient reported as having triggered the decision to initiate a systemic antifungal therapy. **Table E4.** Factors in favor of the prescription of echinocandin agent at SAT initiation (multivariate analysis). **Table E5.** Comparison of patients with initially vs. ultimately proven invasive candidiasis. **Table E6.** Comparison of patients with invasive candidiasis vs. without invasive candidiasis.
