# Supplementary material for: Systemic antifungal therapy for proven or suspected invasive candidiasis: the AmarCAND 2 study
Source: Ann Intensive Care. 2016 Jan 8;6:2. doi: 10.1186/s13613-015-0103-7 (PMC4705061; doi:10.1186/s13613-015-0103-7)
Supplement: Supplementary file 1 — 10.1186/s13613-015-0103-7 Table E1. Characteristics of patients with proven and suspected invasive candidiasis at ICU admission. Table E2. Rate of proven versus suspected invasive candidiasis enrolled by center according to center characteristics. Table E3. Among the 544 ICU patients with SAT for suspicion of invasive candidiasis, characteristics of patient reported as having triggered the decision to initiate a systemic antifungal therapy. Table E4. Factors in favor of the prescription of echinocandin agent at SAT initiation (multivariate analysis). Table E5. Comparison of patients with initially vs. ultimately proven invasive candidiasis. Table E6. Comparison of patients with invasive candidiasis vs. without invasive candidiasis. [file 13613_2015_103_MOESM1_ESM.docx]

**Electronic Supplement Material** to the article entitled

“**Systemic antifungal therapy for proven or suspected invasive candidiasis in French intensive care units: the AmarCAND 2 study“**

**C*linical data collection***

Data were collected for each patient using an electronic case report form (eCRF).

At study inclusion, the following patient data at admission were retrospectively extracted from their files and entered in the eCRF: demographics; severity scores (Simplified Acute Physiology score II (SAPS II) and Sequential Organ Failure Assessment (SOFA) score); medical history, including chronic cardiovascular, neurological, hormonal, renal and lung diseases, congenital or acquired immunodeficiency disorders, malignant solid tumor or hemopathy; surgery in last 3 months and type of surgery; reason for ICU admission. In addition, the following data were collected at the SAT initiation date: date of SAT initiation (D0); temperature; severity scores (SAPS II and SOFA score); presence of septic shock or severe sepsis; use of vasoactive agents; mechanical ventilation; central venous catheter or urinary catheter; renal replacement therapy (hemodialysis or hemofiltration); parenteral nutrition; antibacterial therapy (ongoing at SAT initiation), including its start date and antibacterial agents used; blood transfusion (red cells or platelets); recent surgery, within 3 months before, or during, ICU stay (date, site, cleanliness level, programmed/emergency); local laboratory test results (including procalcitonin, C-reactive protein and creatinine blood levels). Immunodeficiency was defined as patients having undergone a bone marrow transplant, or patients with malignant hemopathy, or patients having undergone a solid organ transplant more than 15 days prior to SAT initiation and treated with immunosuppressive agents, or patients diagnosed with AIDS, patients with auto-immune connective tissue disease, and patients treated with high dose steroids (> 0.5 prenidsolone or equivalent for at least one month). Regarding the IC, in case of PIC, the following data were recorded: date of the positive sample, sample type (blood culture, peritoneal fluid [surgical or percutaneous sample], and other sites), nature of yeast isolated, date of the following results: sample positive with yeast, yeast species identification, antifungal susceptibility profile. In case of SIC, all the data having contributed to the decision of initiating the SAT were recorded: known risk factors for IC, such as known *Candida* colonization (number and nature of sites), total parenteral nutrition, recent surgery, broad spectrum antibacterial therapy, central venous catheter, mechanical ventilation, therapy with steroids, immunosuppressive therapy, anticancer therapy, acute renal failure, renal replacement therapy, severe sepsis or septic shock, high fever despite antimicrobial treatment, acute pancreatitis, underlying diseases such as chronic renal insufficiency and non-culture based mycological tests results [[1](#_ENREF_1)]. *Candida* colonization data was based on investigator declaration. The definition was the presence of *Candida* spp. in any sample taken from different body sites (nose, throat, urine, feces, skin, or bronchi).

The other care procedures associated to SAT initiation that aimed to cure the IC were recorded. Measures to control sources of IC, such as the removal of central venous catheter in candidemia or any surgical procedure in cIAI, were collected.

If yeasts were isolated from usually sterile samples, the number of species was recorded; for each isolate, the species, the body site, the sampling date, the identification date and the antifungal susceptibility profile were recorded.

Information on the SAT was also recorded: single or combined SAT; drug(s) prescribed; daily dose on days 1 to 4. If antibacterial agents were co-administered with the SAT, their nature was recorded, whether it was a therapy initiation or a modification of a prior treatment, and the bacteriological tests results.

In case of PIC, the IC management data were recorded, such as the number of blood cultures and the dates of the last positive blood culture and of the first negative blood culture; the results of the non culture-based blood tests; the imaging examinations and their results; and whether a fundoscopy was performed.

In case of SAT modification, the nature, the date and the reason of the change were recorded. Among the proposed reasons, the investigator could select whether it was based on positive bacteriological results making very unlikely the diagnosis of IC, or based on mycological results requiring adapting the SAT, or due to patient worsening, or due to an adverse event. In case of a new SAT, the starting date, the agent name and dose for the first four days, and whether it was a single agent or a combined therapy were recorded.

The clinical outcome of the patient was recorded. To define cure and failure, we used the criteria proposed by Reboli *et al* [[2](#_ENREF_2)]. Cure was considered if there was both clinical success (resolution of signs and symptoms of invasive candidiasis and no need for additional systemic antifungal therapy) and microbiologic success (eradication of *Candida* species present at baseline, or their presumed eradication, if culture data were not available for a patient with a successful clinical response). Failure was defined as no significant improvement in signs and symptoms, or death due to invasive candidiasis, or persistent or recurrent candidiasis, or a new *Candida* infection. When outcome was neither a failure neither a success, it was considered as not determined. We also recorded SOFA score at D7, SAT stop date, the date of ICU discharge and patient’ status at this date, and the date of hospital discharge and patient’ status at this date, as was the patient’ status at D28.

***Doses of antifungal agents***

At SAT initiation, the median doses of fluconazole, voriconazole, caspofungin and micafungin were 800 mg, 800 mg, 70 mg and 100 mg, respectively. On days 2 to 4, doses were 400 mg, 400 mg, 50 mg and 100 mg, respectively.

***Factors associated with echinocandin prescription***

A multivariate analysis was conducted to analyze the factors associated with echinocandin prescription. Variables with a p-value of 0.2 in an univariate analysis were introduced in a multivariate logistic regression. The selected variables were the followings: (1) at ICU admission: SAPSII, gender, BMI, presence of comorbidities, type of admission (medical reasons *vs*. other), surgery before ICU admission, interval from hospitalization; (2) at SAT initiation: parenteral nutrition, presence of bacteria, septic shock, antibacterial therapy, red blood cell transfusion, creatinine, central venous catheter, proven IC (*vs*. suspected). The final model showed that a proven invasive candidiasis, septic shock, SAPSII score greater than 46, ICU admission for medical reasons, a creatinine greater than 103 µmol/L, and the presence of a central venous catheter increased significantly the probability of echinocandin prescription (see ***Table E4***).

***Table E1***: Characteristics of patients with proven and suspected invasive candidiasis at ICU admission

| Characteristics | Proven invasive candidiasis | Suspected invasive candidiasis | p-value |
| --- | --- | --- | --- |
|  | N = 291 | N = 544 |  |
| Ethnic origin |  |  | 0.34 |
| Caucasian | 267 (92.1) | 513 (94.5) |  |
| African | 19 (6.6) | 21 (3.9) |  |
| Asian | 2 (0.7) | 3 (0.6) |  |
| Hispanic | 2 (0.7) | 6 (1.1) |  |
| Severity scores at admission to ICU |  |  |  |
| SAPS II | 53.1 ± 19.0 | 51.0 ± 18.0 | 0.08 |
| SOFA score | 8.5 ± 4.2 | 8.0 ± 4.1 | 0.046 |
| Underlying disease |  |  |  |
| *Cardiovascular disease* |  |  |  |
| myocardial infarction | 26 (8.9) | 44 (8.1) | 0.67 |
| congestive heart failure | 25 (8.6) | 43 (7.9) | 0.73 |
| peripheral vascular disease | 46 (15.8) | 83 (15.3) | 0.84 |
| *Immunosuppression* |  |  |  |
| blood disorder/bone marrow transplant | 12 (4.0) | 39 (7.2) | 0.08 |
| solid organ transplant >15 days | 7 (2.4) | 22 (4.0) | 0.24 |
| AIDS | 6 (2.1) | 9 (1.7) | 0.79 |
| connective tissue disease | 5 (1.7) | 7 (1.3) | 0.76 |
| corticosteroid treatment | 11 (3.8) | 26 (4.8) | 0.60 |
| *Gastroenterologic disease* |  |  |  |
| moderate to severe liver disease | 13 (4.5) | 26 (4.8) |  |
| mild liver disease | 12 (4.1) | 25 (4.6) |  |
| ulcer disease | 32 (11.0) | 46 (8.5) | 0.26 |
| *Neurological disease* |  |  |  |
| hemiplegia | 10 (3.4) | 6 (1.1) | 0.03 |
| dementia | 2 (0.7) | 10 (1.8) | 0.23 |
| stroke | 18 (6.2) | 27 (5.0) | 0.52 |
| *Endocrinological disease* |  |  |  |
| diabetes with target-organ damage | 24 (8.3) | 36 (6.6) | 0.40 |
| diabetes without target-organ damage | 32 (11.0) | 62 (11.4) | 0.85 |
| Other conditions |  |  |  |
| moderate to severe renal disease | 61 (21.0) | 73 (13.4) | 0.005 |
| chronic lung disease | 44 (15.1) | 88 (16.2) | 0.69 |
| solid malignant tumor | 71 (24.4) | 120 (22.1) | 0.44 |
| Red blood cell transfusion in ICU | 163 (56.0) | 289 (53.1) | 0.42 |
| Platelet transfusion in ICU | 60 (20.6) | 119 (21.9) | 0.67 |
| Surgery in the last three months | *114 (39.2)* | *253 (46.5)* | *0.04* |
| Other data at SAT initiation |  |  |  |
| C-reactive protein (mg/L) | 165.9 ± 109.4 | 173.1 ± 122.6 | 0.67 |
| Creatinine (µmol/L) | 145.8 ± 109.6 | 132.1 ± 96.9 | 0.13 |

The results are given as n (%) or mean ± SD, when indicated.

SAT: systemic antifungal therapy; ICU: intensive care unit; SOFA: sequential organ failure assessment; SAPS II: simplified acute physiology score.

***Table E2***: Rate of proven versus suspected invasive candidiasis enrolled by center according to center characteristics

|  | Proven invasive candidiasis  N=291 | Suspected invasive candidiasis  N=544 | p-value |
| --- | --- | --- | --- |
| Center (>1140 beds) | 121 (41.6) | 238 (43.8) | 0.56 |
| University hospital | 216 (74.2) | 415 (76.3) | 0.55 |
| Type of Intensive Care Unit |  |  | 0.06 |
| Polyvalent | 173 (59.45) | 317 (58.27) |  |
| Medicine | 40 (13.8) | 85 (15.6) |  |
| Surgery | 65 (22.3) | 134 (24.6) |  |
| Specialized | 13 (4.5) | 8 (1.5) |  |
| Number of included patients by center |  |  | <0.01 |
| 1 – 10 | 123 (42.3) | 158 (29.0) |  |
| 11 – 22 | 113 (38.8) | 166 (30.5) |  |
| >22 | 55 (18.9) | 220 (40.4) |  |

The results are given as n (%).

***Table E3***: Among the 544 ICU patients with SAT for suspicion of invasive candidiasis, characteristics of patient reported as having triggered the decision to initiate a systemic antifungal therapy

| Characteristics of patients with SAT for suspected invasive candidiasis | N (%) |
| --- | --- |
| **Clinical conditions** |  |
| Acute renal failure | 147 (27.0) |
| Underlying chronic renal failure | 47 (8.6) |
| Central venous catheter present | 385 (70.8) |
| Severe sepsis or septic shock | 383 (70.4) |
| Invasive mechanical ventilation ongoing | 328 (60.3) |
| Recent surgery (within 3 months before or during ICU stay) | 277 (50.9) |
| High severity scores^§^ | 198 (36.4) |
| Total parenteral nutrition | 162 (29.8) |
| Continuous renal replacement therapy | 126 (23.2) |
| Current corticosteroid therapy (including hydrocortisone hemisuccinate) | 113 (20.8) |
| Recent (<1 month) or current cancer chemotherapy | 47 (8.6) |
| Current immunosuppressive treatment | 37 (6.8) |
| Acute pancreatitis | 25 (4.6) |
| **Microbiological criteria** |  |
| Known Candida spp. colonization  Unifocal colonization  Multiple site colonization | 206 (37.9)  92 (16.9)  114 (21.0) |
| Prolonged broad-spectrum antibiotics (current or in the previous 10 days) | 300 (55.2) |
| Persistent hyperthermia despite antibacterial agents | 196 (36.0) |
| Non culture-based laboratory tests (positive (1-3)-β-D-glucan, mannan antigens/antibodies and/or *Candida* PCR) | 20 (3.7) |

The results are given as n (%).

§: according to investigator’s judgment

***Table E4***: Factors in favor of the prescription of echinocandin agent at SAT initiation (multivariate analysis)

| Factors with OR > 1 | OR | 95%CI | | | p-value |
| --- | --- | --- | --- | --- | --- |
| Proven invasive candidiasis | 1.933 | 1.41 |  | 2.654 | <.01 |
| Septic shock | 1.754 | 1.28 |  | 2.401 | <.01 |
| SAPS II (>46) | 1.391 | 1.02 |  | 1.903 | 0.04 |
| ICU admission with medical diagnosis | 1.807 | 1.35 |  | 2.42 | <.01 |
| Creatinine (>103 µmol/L) | 1.546 | 1.15 |  | 2.083 | <.01 |
| Central venous catheter | 2.857 | 1.27 |  | 6.447 | 0.01 |

SAT: systemic antifungal therapy; ICU: intensive care unit; SAPS II: simplified acute physiology score; OR: Odd ratio.

***Table E5***: Comparison of patients with initially vs. ultimately proven invasive candidiasis

| Patients’ characteristics | Initially proven invasive candidiasis  (N=291) | Ultimately proven invasive candidiasis  (N=112) | p-value |
| --- | --- | --- | --- |
| **ICU admission** |  |  |  |
| Age | 62.6 [52.9 ; 74.1] | 63.1 [54.5 ; 72.3] | 0.78 |
| Male gender | 185 (63.6) | 69 (61.6) | 0.71 |
| BMI | 26.3 [22.5 ; 31.5] | 24.9 [22.2 ; 28.9] | 0.06 |
| Previous duration of hospital stay (days) | 2 [0 ; 10] | 1 [0 ; 7] | 1 |
| SAPSII | 45 [33 ; 59] | 48 [38 ; 61] | 0.08 |
| SOFA Score | 9 [6 ; 11] | 8 [5 ; 10] | 0.04 |
| **SAT initiation** |  |  |  |
| Duration of ICU stay before initial SAT (days) | 5 [2 ; 14] | 1 [0 ; 8] | <.01 |
| Body temperature (°C) | 37.7 [37 ; 38.5] | 38 [36.7 ; 38.6] | 0.58 |
| SOFA score | 7 [3 ; 11] | 7 [5 ; 10] | 0.27 |
| Septic shock | 120 (41.2) | 62 (55.4) | 0.01 |
| Severe sepsis | 118 (40.5) | 47 (42) | 0.8 |
| Invasive mechanical ventilation | 196 (67.4) | 92 (82.1) | <.01 |
| Central venous catheter | 278 (95.5) | 109 (97.3) | 0.41 |
| Urinary catheterization | 280 (96.2) | 108 (96.4) | 0.92 |
| Hemodialysis or hemodiafiltration | 92 (31.6) | 29 (25.9) | 0.26 |
| Total parenteral nutrition | 149 (51.2) | 50 (44.6) | 0.24 |
| Antimicrobial treatment | 268 (92.1) | 97 (86.6) | 0.09 |
| Corticosteroid treatment | 55 (18.9) | 27 (24.1) | 0.24 |
| Red blood cell transfusion | 163 (56) | 57 (50.9) | 0.35 |
| Platelet transfusion | 60 (20.6) | 25 (22.3) | 0.71 |
| Surgery just before or during ICU stay | 197 (67.7) | 87 (77.7) | 0.05 |
| Creatinine (µmol/L) | 108 [66 ; 197] | 99.5 [56 ; 176] | 0.16 |

The results are given as n (%) or median (min; max), when indicated.

SAT: systemic antifungal therapy; ICU: intensive care unit; SOFA: sequential organ failure assessment; SAPS II: simplified acute physiology score

***Table E6***: Comparison of patients with invasive candidiasis vs. without invasive candidiasis.

| Patients’ characteristics | Patients with IC  (N=403) | Patients without IC  (N=432) | p-value |
| --- | --- | --- | --- |
| **ICU admission** |  |  |  |
| Age | 62.7 [53 ; 73.8] | 63.8 [53.2 ; 73] | 0.89 |
| Male | 254 (63) | 268 (62) | 0.77 |
| Previous duration of hospital stay (days) | 2 [0 ; 9] | 2 [0 ; 10] | 0.4 |
| SAPSII | 46 [34 ; 59] | 48 [36 ; 62] | 0.04 |
| SOFA Score | 8 [6 ; 11] | 8 [5 ; 11] | 0.39 |
| **SAT initiation** |  |  |  |
| Duration of ICU stay before initial SAT (days) | 4 [1 ; 12] | 5 [1 ; 12] | 0.08 |
| Body temperature | 37.8 [37 ; 38.5] | 38 [37 ; 38.5] | 0.42 |
| SOFA score | 7 [4 ; 10] | 8 [5 ; 11] | <.01 |
| Septic shock | 182 (45.2) | 268 (62) | <.01 |
| Severe sepsis | 165 (40.9) | 156 (36.1) | 0.15 |
| Invasive mechanical ventilation | 288 (71.5) | 366 (84.7) | <.01 |
| Central venous catheter | 387 (96) | 417 (96.5) | 0.7 |
| Urinary catheterization | 388 (96.3) | 408 (94.4) | 0.21 |
| Hemodialysis or hemodiafiltration | 121 (30) | 131 (30.3) | 0.93 |
| Total parenteral nutrition | 199 (49.4) | 185 (42.8) | 0.06 |
| Antimicrobial treatment | 365 (90.6) | 384 (88.9) | 0.42 |
| Corticosteroid treatment | 82 (20.3) | 124 (28.7) | <.01 |
| Red blood cell transfusion | 220 (54.6) | 232 (53.7) | 0.8 |
| Platelet transfusion | 85 (21.1) | 94 (21.8) | 0.81 |
| Surgery just before or during ICU stay | 284 (70.5) | 254 (58.8) | <.01 |
| Creatinine (µmol/L) | 106 [63 ; 194] | 104.5 [65 ; 168] | 0.45 |

The results are given as n (%) or median (min; max), when indicated.

IC: invasive candidiasis; SAT: systemic antifungal therapy; ICU: intensive care unit; SOFA: sequential organ failure assessment; SAPS II: simplified acute physiology score

**REFERENCES**

1. Guery BP, Arendrup MC, Auzinger G, Azoulay E, Borges Sa M, Johnson EM et al. Management of invasive candidiasis and candidemia in adult non-neutropenic intensive care unit patients: Part I. Epidemiology and diagnosis. Intensive care medicine. 2009;35(1):55-62. doi:10.1007/s00134-008-1338-7.

2. Reboli AC, Rotstein C, Pappas PG, Chapman SW, Kett DH, Kumar D et al. Anidulafungin versus fluconazole for invasive candidiasis. The New England journal of medicine. 2007;356(24):2472-82. doi:10.1056/NEJMoa066906.
